# Oral Treatment with the Ghrelin Receptor Agonist HM01 Attenuates Cachexia in Mice Bearing Colon-26 (C26) Tumors

**DOI:** 10.3390/ijms18050986

**Published:** 2017-05-05

**Authors:** Fabienne O. Villars, Claudio Pietra, Claudio Giuliano, Thomas A. Lutz, Thomas Riediger

**Affiliations:** 1 Institute of Veterinary Physiology, University of Zurich, 8057 Zurich, Switzerland; fabienne.villars@vetphys.uzh.ch (F.O.V.); tomlutz@vetphys.uzh.ch (T.A.L.); 2 Zurich Center for Human Integrative Physiology, University of Zurich, 8057 Zurich, Switzerland; 3 Helsinn Healthcare SA, 6912 Pazzallo–Lugano, Switzerland; claudio.pietra@helsinn.com (C.P.); claudio.giuliano@helsinn.com (C.G.)

**Keywords:** muscle wasting, bone mineral density, food intake, energy expenditure, macrophage-inhibitory cytokine-1 (MIC-1)/growth differentiation factor-15 (GDF15)

## Abstract

The gastrointestinal hormone ghrelin reduces energy expenditure and stimulates food intake. Ghrelin analogs are a possible treatment against cancer anorexia-cachexia syndrome (CACS). This study aimed to investigate whether oral treatment with the non-peptidergic ghrelin receptor agonist HM01 counteracts CACS in colon-26 (C26) tumor-bearing mice. The C26 tumor model is characterized by pronounced body weight (BW) loss and muscle wasting in the absence of severe anorexia. We analyzed the time course of BW loss, body composition, muscle mass, bone mineral density, and the cytokines interleukin-6 (IL-6) and macrophage-inhibitory cytokine-1 (MIC-1). Moreover, we measured the expression of the muscle degradation markers muscle RING-finger-protein-1 (MuRF-1) and muscle atrophy F-box (MAFbx). After tumor inoculation, MIC-1 levels increased earlier than IL-6 and both cytokines were elevated before MuRF-1/MAFbx expression increased. Oral HM01 treatment increased BW, fat mass, and neuronal hypothalamic activity in healthy mice. In tumor-bearing mice, HM01 increased food intake, BW, fat mass, muscle mass, and bone mineral density while it decreased energy expenditure. These effects appeared to be independent of IL-6, MIC-1, MuRF-1 or MAFbx, which were not affected by HM01. Therefore, HM01 counteracts cachectic body weight loss under inflammatory conditions and is a promising compound for the treatment of cancer cachexia in the absence of severe anorexia.

## 1. Introduction

The cancer anorexia-cachexia syndrome (CACS) is characterized by reduced dietary intake, tissue wasting, severe body weight loss, and weakness [1]. Inflammatory signaling molecules (e.g., cytokines) released by the tumor itself or the host immune system in response to the malignancy are important mediators of CACS [2]. CACS affects up to 80% of patients with advanced cancer. Regardless of the type of malignancy, cancer patients suffering from CACS show poor responsiveness to anti-cancer treatments, reduced quality of life and increased mortality [3,4,5]. Despite the high prevalence of CACS in cancer patients, to date no effective treatment exists to counteract this condition. Therapeutic approaches against CACS should stimulate energy intake and anabolism to defeat catabolic processes and increase lean mass and body weight [5]. While a few drugs have been developed to improve appetite and increase body weight, none of these drugs effectively increased lean body mass [6]. Furthermore, commonly used appetite stimulating drugs such as corticoids or progestational agents have substantial adverse side effects [7,8].

The gastrointestinal hormone ghrelin stimulates food intake and growth hormone (GH) secretion [9,10] which mediates ghrelin’s anabolic action via insulin-like growth factor-1 (IGF-1) signaling [11,12]. Thus, ghrelin and ghrelin analogs are considered as a possible treatment for CACS [13]. Ghrelin is a high-affinity ligand for the growth hormone secretagogue receptor (GHS-R) and is mainly secreted by the stomach during fasting and shortly before meals [11]. Ghrelin administration in rodents increases food intake and body weight gain via the hypothalamic arcuate nucleus (Arc) [14]. The Arc is a key regulator of food intake and energy homeostasis [15] containing a high density of GHS-R [16]. Various electrophysiological and immunohistological studies demonstrated excitatory effects of ghrelin on Arc neurons [17,18]. Ghrelin also activates muscle cell differentiation in myoblasts [19], suppresses the expression of inflammatory cytokines [20,21] and regulates energy homeostasis [22]. Ghrelin is thought to counteract muscle degradation by IGF-1-dependent inhibition of proteolytic factors such as the E3 ubiquitin ligases muscle RING-finger protein-1 (MuRF-1) and muscle atrophy F-box (MAFbx)/atrogin-1 [12]. Although previous human clinical trials [23,24,25] and studies in rodent cancer models [26,27,28,29,30,31] confirmed therapeutic effectiveness of ghrelin or ghrelin analogues as anti-CACS treatment, currently no ghrelin-based drug is approved.

We recently characterized the effects of the GHS-R agonist HM01 on food intake and body weight gain in the rat Morris-7777 hepatoma tumor model [27]. HM01 is a small molecule compound with high receptor binding affinity, brain penetration, and a long plasma half-life time [32]. Tumor-bearing (TB) rats chronically treated with subcutaneous infusions of HM01 showed 30% higher food intake as well as increased lean and fat mass compared to vehicle-treated TB rats. The Morris-7777 hepatoma tumor model is characterized by anorexia and the absence of pro-inflammatory cytokines such as interleukin-6 (IL-6) or tumor necrosis factor-α (TNF-α) but elevated macrophage-inhibitory cytokine-1 (MIC-1) [33]. While MIC-1 emerged as an important mediator of CACS, it needs to be elucidated whether HM01 also counteracts CACS under cancer conditions that are characterized by pro-inflammatory cytokines, which are often elevated in cancer patients [34]. In our studies, we therefore used the mouse colon-26 (C26) tumor model. The C26 tumor model is well established and characterized by a profound increase of pro-inflammatory cytokines such as IL-6 and TNF-α, severe body weight loss and only a moderate decrease in food intake compared to other CACS tumor models [35]. Hence, it was a major aim of our study to explore whether HM01 counteracts cachectic body weight loss, when cachexia develops without profound anorexia. Since HM01 has been developed for therapeutic use, we aimed to confirm the effectiveness of HM01 during chronic oral administration. First, we tested the effects of chronic oral HM01 treatment on food intake, body weight, body composition and c-Fos expression of Arc neurons in non tumor-bearing (NTB) mice. Second, we investigated whether HM01 improves energy balance in C26 TB mice. While most CACS studies only focus on lean and fat mass, we also analyzed bone mineral density because bone degradation is a clinically relevant problem in cancer patients [36]. Finally, we investigated the effect of HM01 on energy expenditure, pro-inflammatory cytokine levels and the expression of the muscle degradation markers MuRF-1 and MAFbx in C26 TB mice.

## 2. Results

### 2.1. Effects of HM01 on Food Intake, Body Weight, and Neuronal Activation in Non-Tumor-Bearing Mice

**Figure 1:**
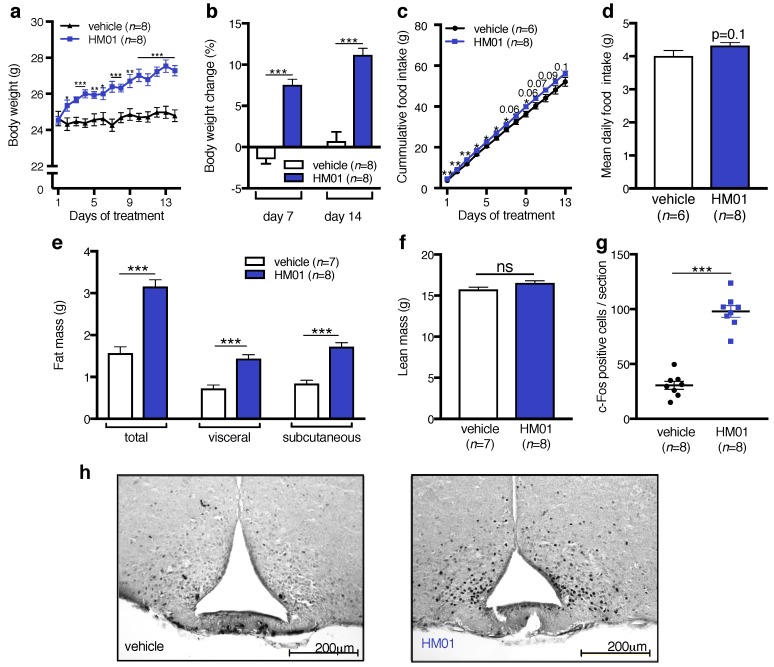
Effects of oral HM01 treatment (10 mg/kg/day) on food intake, body weight, and neuronal activation in non tumor-bearing mice. (**a**) HM01-treated mice increased body weight from the second day of treatment until the end of experiment; (**b**) Body weight gain was significantly increased after 7 and 14 days of HM01 treatment compared to vehicle-treated animals; (**c**) HM01 stimulated total food intake over the 14-day treatment period (**d**) but had no effect on mean daily food intake; (**e**) Higher body weight was mainly due to increased total, visceral and subcutaneous fat mass in HM01 vs. vehicle-treated animals; (**f**) There was no difference in lean mass between the groups. Data in e and f were acquired with computed tomography at the end of experiment; (**g**) HM01-treated animals show increased neuronal activation in the arcuate nucleus. When perfused 2 h after the treatment at day 14, they showed significantly more c-Fos positive cells; (**h**) Representative pictures of the arcuate nucleus in vehicle and HM01-treated mice stained against c-Fos. Data displayed as mean ± standard error of the mean and analyzed with Student’s *t*-Test. * *p* < 0.05, ** *p* < 0.01, *** *p* < 0.001.

Oral HM01 treatment significantly increased body weight relative to vehicle-treated controls; the body weight difference became significant from the second day of treatment (Figure 1a). HM01-treated animals showed a body weight increase of 7.6 ± 0.7% (vehicle: −1.5 ± 0.6%) after 7 days and 11.2 ± 0.8% (vehicle: 0.7 ± 1.1%) after 14 days (Figure 1b). HM01 also stimulated cumulative food intake although significance was not reached during the last 4 days of treatment (Figure 1c). In total, HM01-treated mice consumed 4.1 g more food than controls during the 14-day treatment period. HM01-treated mice consumed 8.1 ± 2.3% more as a daily average which was however not significant (Figure 1d). The body weight gain was mainly due to increased total (3.2 ± 0.2 g vs. 1.6 ± 0.2 g, *p* < 0.001), visceral (1.4 ± 0.1 g vs. 0.7 ± 0.1 g, *p* < 0.001 ) and subcutaneous (1.7 ± 0.1 g vs. 0.8 ± 0.1 g, *p* < 0.001) fat mass (Figure 1e); HM01 treatment had no effect on lean mass in NTB mice (Figure 1f). At the end of the experiment, HM01-treated mice displayed significantly higher neuronal activation of the Arc as measured by c-Fos expression (Figure 1g,h).

### 2.2. Cancer-Induced Effects on Body Weight, Food Intake, Metabolism, and Locomotor Activity

**Figure 2:**
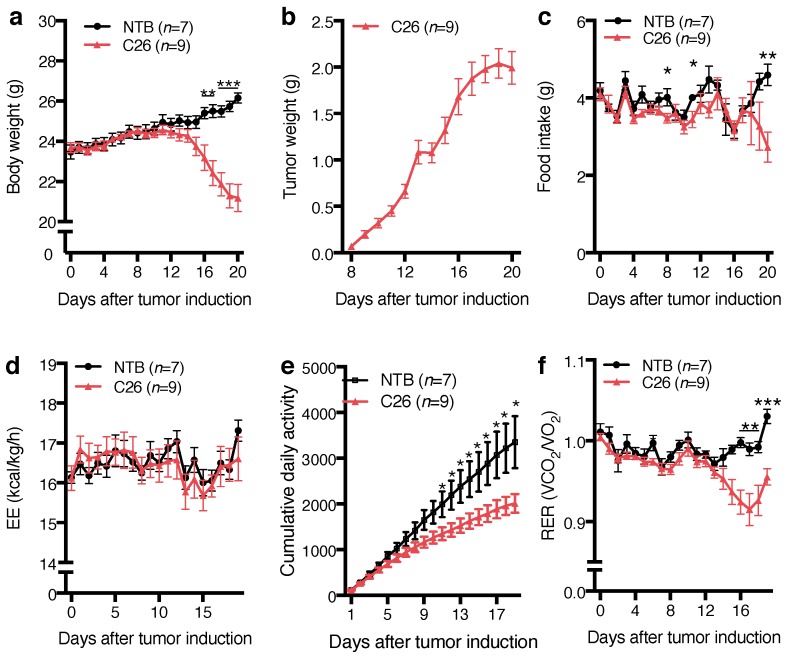
Effects of tumor growth on body weight, food intake, metabolism and locomotor activity; comparison between non tumor-bearing animals (NTB) and colon-26 tumor-bearing mice (C26). (**a**) C26 mice displayed significant body weight loss 16 days after tumor induction. Body weight was corrected for tumor weight; (**b**) Tumors grew continuously from the first day after tumor induction. From day 8, tumor size was measured and tumor weight calculated from tumor dimensions using the following equation: 0.75 × 0.5 × (height × width${}^{2}$); 1 cm${}^{3}$ = 1 g; (**c**) Daily food intake was similar in NTB und C26 mice and only significantly different on days 8, 11 and 20; (**d**) No difference was observed in energy expenditure (EE) rate (kcal/kg/day) in tumor-bearing (TB) compared to NTB mice; (**e**) Total daily activity in C26 mice was reduced from day 11 after tumor induction; (**f**) Respiratory exchange ratio (RER) was significantly lower in C26 mice from 15 days after tumor induction. Data displayed as mean ± standard error of the mean and analyzed with Student’s *t*-Test. * *p* < 0.05, ** *p* < 0.01, *** *p* < 0.001.

TB animals started to lose body weight (corrected for tumor weight) from day 12, reaching statistical significance 16 days after tumor induction (Figure 2a). Tumors were first measurable after 8 days and reached a mass of 2.0 ± 0.2 g 20 days after tumor cell inoculation (Figure 2b). At the end of experiment, TB mice weighed 5 g less than NTB controls. Food intake was unaffected for most time points of the experiment, except on days 8, 11 and 20 when TB animals consumed significantly less food compared to NTB mice (Figure 2c). TB animals displayed a similar metabolic rate (kcal/kg/h) as NTB mice (Figure 2d), although their locomotor activity was significantly reduced (Figure 2e). TB mice showed significantly decreased respiratory exchange ratio (RER) from day 15 after tumor induction (Figure 2f), indicating a metabolic shift towards fat oxidation.

### 2.3. Body Composition, Inflammatory Cytokines, and Muscle Degradation Markers at Different Time Points during C26 Tumor Growth

**Figure 3:**
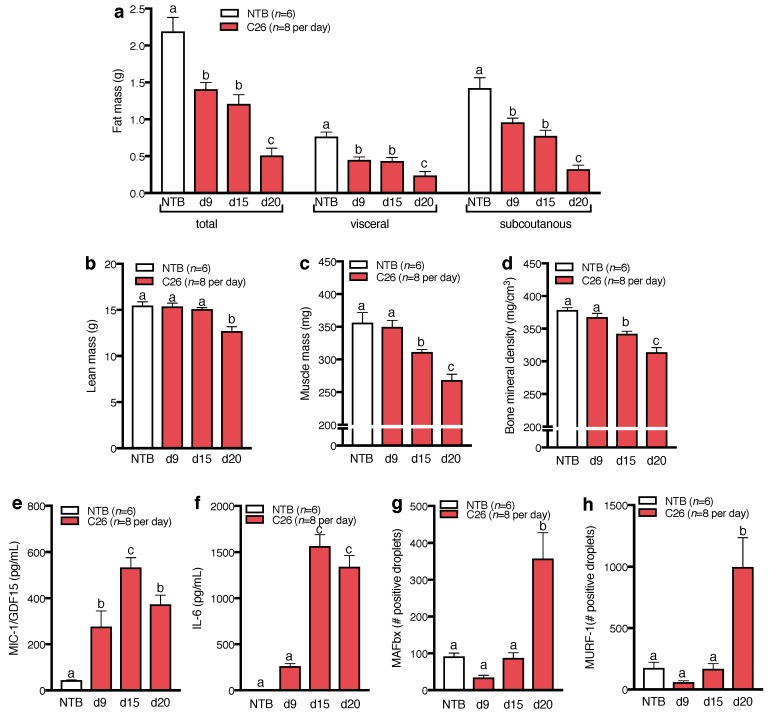
Time course of body composition, cytokines and muscle proteolytic enzymes in C26 tumor-bearing mice compared to non tumor-bearing (NTB) controls. A group of C26 mice was euthanized at 9 (d9), 15 (d15) and 20 (d20) days after tumor induction. (**a**) Total, visceral and subcutaneous fat mass decreased from 9 days after tumor induction in C26 tumor-bearing mice compared to NTB controls; (**b**) Overall lean mass decrease was detectable 20 days after tumor induction; (**c**) Muscle mass of the right hind limb (sum of tibias anterior, gastrocnemius, soleus and quadriceps) of C26 mice was reduced from day 15; (**d**) C26 also showed progressive decrease of bone mineral denisty from 15 days after tumor induction; (**e**) interleukin-6 (IL-6) plasma levels were increased 15 and 20 days after tumor induction compared to NTB and (**f**) macrophage-inhibitory cytokine-1 (MIC-1) levels were elevated 9, 15 and 20 days after tumor induction in C26 mice; (**g**) The E3 ubiquitin ligases muscle atrophy F-box (MAFbx) and (**h**) muscle RING finger-1 (MURF-1) were increased after 20 days as quantified by digital droplet PCR and displayed as positive droplets per 0.75 ng of complementary DNA (cDNA) input. Data displayed as mean ± standard error of the mean and analyzed with one-way ANOVA followed by Turkey post-hoc test. Different letters indicate significant difference, *p* < 0.05.

A separate experiment was conducted to analyze the time courses of body composition, muscle mass, bone mineral density, inflammatory cytokines and E3 ligases MuRF-1 and MAFbx in TB vs. NTB mice. TB mice showed progressive loss of fat mass (Figure 3a). No changes in lean mass occurred relative to controls until day 15, while TB mice had reduced lean mass on day 20 (Figure 3b). Reduced hind limb muscle mass was evident 15 days after tumor cell inoculation and declined further until day 20 (Figure 3c). TB mice also showed reduced bone mineral density from day 15, which further decreased until day 20 (Figure 3d). Plasma levels of IL-6 were increased in TB mice at days 15 and 20 after tumor cell inoculation (Figure 3e). Interestingly, MIC-1 levels were already significantly increased from day 9 (Figure 3f). Although significant loss of muscle mass was detected 15 days after tumor induction, the E3 ubiquitin ligases MAFbx (Figure 3g) and MuRF-1 were only increased 20 days after tumor cell inoculation (Figure 3h).

### 2.4. Effects of HM01 on Food Intake and Cachectic Body Weight Loss in C26 Tumor-Bearing Mice

**Figure 4:**
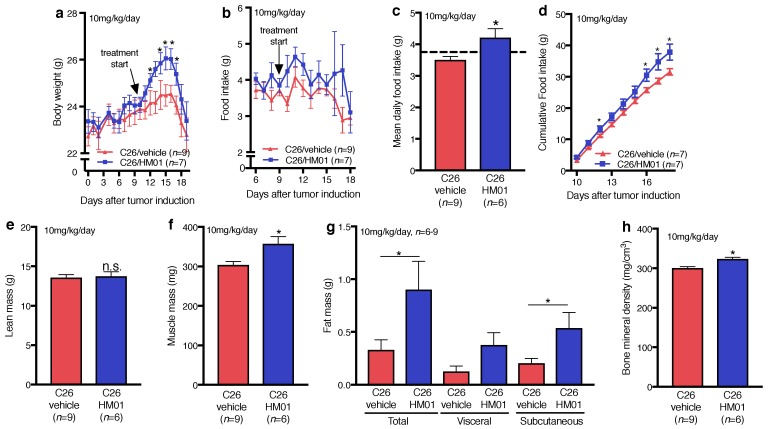
Effects of HM01 treatment on body weight and body composition in C26 tumor-bearing (C26) mice, 1x daily treatment with 10 mg/kg HM01 or vehicle. (**a**) HM01 increased body weight reaching significant difference on days 14–17; (**b**) Although food intake was not significantly different in HM01-treated mice on single days compared to vehicle controls, (**c**) HM01 animals displayed higher mean daily food intake during the treatment period. The dashed line indicates average food intake before the treatment (days 0–9 after tumor induction); (**d**) The cumulative food intake was significantly higher on day 12 and 16–18; (**e**) HM01 administration had no effects on over all lean mass; (**f**) However, muscle mass was significantly higher in HM01-treated mice; (**g**) Total and subcutaneous fat mass was significantly increased following HM01 treatment compared to the vehicle treatment, while visceral fat mass difference was not statistically significant; (**h**) In addition, bone mineral density increased with HM01 treatment. Data displayed as mean ± standard error of the mean and analyzed with Student’s *t*-Test. * *p* < 0.05.

HM01 (10 mg/kg/day) significantly increased body weight in TB animals between 13 and 17 days after tumor induction compared to vehicle-treated TB controls (Figure 4a). However, despite the clear positive effect on body weight development, HM01 treatment did not prevent late- stage body weight loss during the last two days of the experiment (Figure 4a). Food intake was not significantly increased in HM01-treated animals on single days (Figure 4b), but HM01 significantly stimulated food intake averaged across the treatment period (Figure 4c). Compared to vehicle controls, HM01-treated mice consumed 20.2 ± 8.1% more. In total HM01-treated mice consumed 6.3 g more (Figure 4d). The difference in body weight following HM01 administration appeared to be mainly due to higher fat mass (Figure 4g) but not lean mass (Figure 4e), at least when measured by computed tomography (CT) scan. Importantly, however, HM01 significantly increased hind limb muscle mass compared to controls (Figure 4f). Furthermore, the treatment also had a beneficial effect on bone mineral density (Figure 4h).

### 2.5. Effects of HM01 on Cytokines and the E3 Ubiquitin Ligases MuRF-1 and MAFbx in C26 Tumor-Bearing Mice

**Figure 5:**
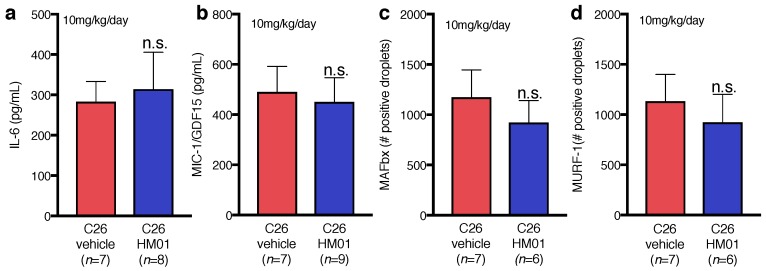
Effects of HM01 (10 mg/kg/day) on cytokines and the E3 ubiquitin ligases in C26 tumor-bearing (C26) mice. (**a**) Interleukin-6 (IL-6) plasma levels were comparable in HM01 and vehicle-treated mice; (**b**) HM01 treatment had also no effect on the plasma levels of the macrophage inhibitory cytokine-1/growth differentiation factor-15 (MIC-1/GDF-15); (**c**,**d**) The gene expression of muscle atrophy F-box (MAFbx) and muscle RING finger-1 (MuRF-1) in HM01-treated mice was similar to mice receiving vehicle as measured by digital droplet PCR. PCR data are expressed as total positive droplets per 0.75 ng of complementary DNA (cDNA) input. Data displayed as mean ± standard error of the mean and analyzed with Student’s *t*-Test.

HM01 treatment had no effects on plasma levels of the inflammatory cytokines IL-6 (Figure 5a) and MIC-1 (Figure 5b). Both cytokines remained highly elevated and not significantly different in both experimental groups (compare Figure 3e–f). HM01-treated TB mice also showed no reduction in the gene expression of MAFbx (Figure 5c) and MuRF-1(Figure 5d).

### 2.6. Effects of HM01 on Metabolic Parameters in C26 Tumor-Bearing Mice

**Figure 6:**
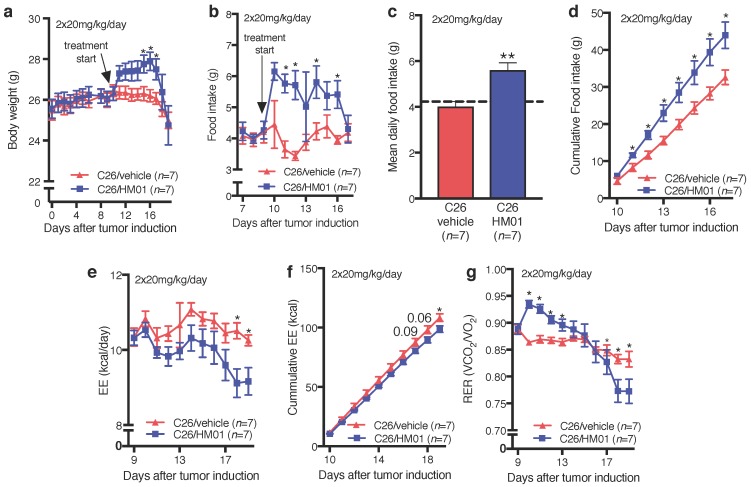
Effects of HM01 (2 × 20 mg/kg/day) on food intake and metabolism. (**a**) HM01 increased body weight in HM01-treated mice leading to a significant difference on days 15–17 after tumor induction; (**b**) Animals treated with HM01 showed significantly higher food intake on days 11, 12, 14 and 16 after tumor inoculation; (**c**) HM01-treated animals also displayed increased mean daily food intake compared to vehicle controls. The dashed line represents the mean daily food intake in the period before HM01 treatment (days 0–9 after tumor induction); (**d**) Total food intake was increased following HM01 treatment from day 11 after tumor cell inoculation until the end of experiment; (**e**) Energy expenditure (EE) was significantly reduced in HM01 compared to vehicle-treated animals on days 18 and 19; (**f**) HM01 also decreased total energy expenditure over the treatment period; (**g**) The respiratory exchange ration (RER) was significantly increased by HM01 administration in the first 4 days of treatment, while it then decreased leading to significantly lower RER on days 18 and 19 compared to vehicle-treated controls. Data displayed as mean ± standard error of the mean and analyzed with Student’s *t*-Test. * *p* < 0.05.

**Figure 7:**
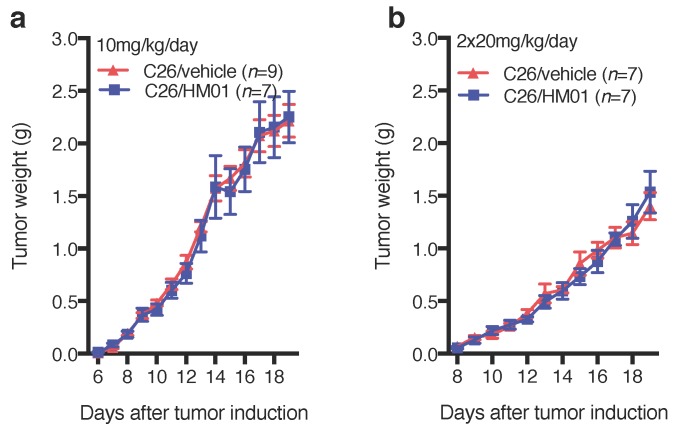
Calculated tumor weight (1 cm${}^{3}$ = 1 g; 0.75 × 0.5 × (height × width${}^{2}$)). (**a**,**b**) Importantly, neither treatment doses had any effect on tumor growth. Data displayed as mean ± SEM and analyzed with Student’s *t*-Test.

We explored the effect of HM01 on energy expenditure and respiratory exchange ratio in TB mice. Since HM01 did not prevent late stage body weight loss in our studies using 10 mg/kg/day (Figure 4a), we increased the dose to 2 × 20 mg/kg/day in this experiment. HM01 administration increased body weight, reaching statistical significance on days 15–17 (Figure 6a). However, similar to the lower dose, HM01 did not prevent severe body weight loss during days 18–19. In contrast to the lower dose, HM01 significantly increased daily food intake in HM01-treated animals on days 11, 12, 14 and 16 after tumor inoculation, which corresponds to treatment days 2, 3, 5 and 7, respectively (Figure 6b). HM01 increased mean daily food intake during the treatment period compared to vehicle-treated animals (Figure 6c). Cumulative food intake was significantly higher in HM01-treated mice between days 10 and 17 (Figure 6d). We were unable to measure food intake during the last two days of experiment due to extreme spillage. HM01 reduced energy expenditure, although statistical significance was only reached on the last two days of the experiment (Figure 6e). Cumulative energy expenditure only differed between the groups on day 19 (Figure 6f). Furthermore, HM01-treated mice showed a significantly higher respiratory exchange ration (RER) for the first 4 treatment days and a lower RER during the last three days (day 17–19) (Figure 6g). Importantly, HM01 treatment did not affect tumor size at both doses at any time during the treatment (Figure 7).

## 3. Discussion

It was a major aim of this study to explore whether oral treatment with the ghrelin receptor agonist HM01 counteracts CACS in a tumor model that is characterized by pronounced cachexia in the absence of severe anorexia. For this purpose, we used the C26 colon carcinoma model in mice. In order to better understand the mechanisms contributing to the progression of CACS, we analyzed the time course of changes in body weight, body composition, muscle mass, bone mineral density, cytokine levels and muscle degradation markers. Among the investigated cytokines, MIC-1 showed the earliest increase during tumor growth, which coincided with the onset of cachectic body weight loss. Interestingly, muscle wasting was evident before the activation of the E3 ubiquitin ligase pathway (MURF-1 and MAFbx), which was upregulated only at late stage cachexia. HM01 increased body weight in NTB mice by increasing fat but not lean mass. Energy intake did not appear to account for this effect because food intake was only marginally increased. HM01-treated NTB mice showed increased neuronal Arc activity at the end of the experiment, indicating that the animal are still sensitive to HM01 after 14 days of chronic treatment. In healthy mice HM01 attenuated tumor-induced loss of body weight, fat mass, muscle mass and bone mineral density. In contrast to NTB mice, HM01 increased energy intake in TB mice compared to controls. HM01 also increased body weight, fat mass, muscle mass and bone mineral density. Moreover, we demonstrated a reduction of the metabolic rate by HM01 under cancer conditions. These beneficial anti-CACS treatment effects were independent of changes in cytokine or E3 ligase expression levels. Our findings provide evidence that HM01 overrides inflammatory early and mid stage cachexia that is not primarily caused by anorexia.

Given the orexigenic action of ghrelin [9,14,37] and ghrelin agonists [27,28,38] in rodents, it was unexpected that HM01 did not induce a clear increase in food intake in NTB animals, although HM01-treated mice consumed slightly more food in the first few days of treatment [27]. Therefore, the increase in body weight appears to be mediated by other mechanisms than increased energy intake. The reduction of the metabolic rate is a well documented effect of ghrelin [39,40] promoting body weight gain and fat mass accumulation. Moreover, ghrelin signaling promotes adiposity by increasing lipogenesis and decreasing fat oxidation [41,42]. Both the increase in body weight and neuronal activation in the Arc persisted until the end of the 14-day treatment period. This finding is in line with our recent observations in HM01-treated rats [27], indicating that chronic treatment does not lead to desensitization, which is a therapeutically relevant finding. It was beyond our aims to dissociate central and peripheral effects that contribute to the positive effect of HM01 on energy balance. However, the activation of the Arc confirms a central nervous action of HM01 and is in line with our previous electrophysiological demonstration of an excitatory effect of HM01 on Arc neurons [27].

The C26 tumor model is a frequently used model of cachectic body weight loss [35,43,44,45,46,47,48]. It is characterized by increased levels of inflammatory cytokines including IL-6 [45,49] and severe body weight loss in the absence of an anorectic response [35]. C26 TB mice showed behavioral and metabolic alteration, including a decrease in RER and physical activity. Inactivity and lethargy are well known symptoms of different inflammatory disease and cancer cachexia [35,50] associated malaise. While metabolic rate was not affected by cancer growth, C26 mice might be considered hypermetabolic because their locomotor activity is considerably decreased without a decrease in energy expenditure. Hence, adaptive changes in metabolism that usually occur in healthy animals seemed to be disturbed in C26 mice. Apart from this, indirect calorimetry does not detect changes in anaerobic metabolism, which typically occurs in tumor tissue [51,52] and might also contribute to negative energy balance in TB mice.

The presence of inflammatory cytokines in C26 mice is a major difference to our previous study evaluating the ability of HM01 to attenuate CACS in rats bearing hepatoma Morris-7777 tumors [27]. In order to evaluate the full therapeutic potential and ability to counteract CACS, it is important to use different tumor models reflecting the heterogeneity of CACS in cancer patients. Our study is the first to analyze body composition, cytokines and muscle proteolytic factors in this model at different time points during tumor progression. Apparently, the pathological events contributing to CACS in this tumor model occur in sequence. While MIC-1 levels increases early during tumor progression, increased expression of muscle degradation markers MuRF-1 and MAFbx only takes place at a later stage. While this pathway is considered as a key mediator of cachectic body weight loss [4,12], in our studies cachexia and the reduction in muscle mass occurred before a detectable upregulation of this muscle degradation pathway. It has to be noted, however, that the strong increase in E3 ligase expression appeared to coincide with the severe terminal body weight loss during the last 2–3 days of the cachectic phase. Whether MuRF-1 and MAFbx contribute to this dramatic deterioration remains unknown. Furthermore, a possible trigger that might explain this late stage decline remains to be identified.

Given the absence of a clear orexigenic action in NTB mice, the HM01-dependent increase in energy intake in TB mice appears unexpected. This finding is important because experimental and clinical CACS has been proposed to be characterized by ghrelin resistance [53,54]. Our study does not support this concept because HM01 stimulated food intake in TB mice even stronger than in healthy animals at least with the used doses. Moreover, the HM01-induced feeding response appeared to be dose-dependent although we did not directly compare the energy intake between the two treatment paradigms that were used. While the increased energy intake probably contributed to beneficial effects of HM01 treatment against CACS, energy balance was also positively influenced by decreased energy expenditure. HM01 treatment reduced energy expenditure in C26 TB mice which is in line with a reduction in metabolic rate after ghrelin treatment [39,40]. HM01 increased RER in TB mice indicating reduced fat oxidation and consistent with the preservation of adipose tissue mass. However, HM01-treated mice showed increased RER only during the first days of treatment but lower RER than vehicle controls towards the end of the experiment. Ghrelin-stimulated increase in fat mass has been associated with increased expression of lipogenic enyzmes [41] or lipid retention in white adipose tissue [42]. These mechanisms may also have contributed to the effect of HM01 to increase fat mass in TB and NTB mice.

In addition to its effect on fat mass, HM01 treatment counteracted muscle wasting in C26 mice. Similar to our previous studies in TB rats [27], this effect was only reflected by increased muscle weight, but not by overall lean mass measured by CT scans. Therefore, negative outcomes of CT studies with respect to lean mass should be interpreted cautiously because they do not necessarily exclude positive treatment effects on muscle mass. Muscle mass depends on balanced processes leading to muscle tissue buildup and muscle degradation. According to our findings, we consider it unlikely that the HM01-dependent effect on muscle mass depends on the E3 ligase pathway, because HM01 did not affect MuRF-1/MAFbx gene expression. Although we did not find evidence for a MuRF-1/MAFbx- dependent treatment effect of HM01, previous studies demonstrated a blockade of MuRF-1/MAFbx signaling in tumor-bearing mice following ghrelin treatment [26]. This effect was paralleled or due to a ghrelin-induced suppression of cytokines, which we also did not observe under our experimental conditions. Whether this discrepancy might be related to differences in anti-inflammatory effectiveness between HM01 and the native ligand ghrelin needs to be elucidated. The growth hormone secretagogue receptor 1a (GHS-R1a) is able to activate different intracellular second messenger systems depending on the agonist activating the receptor [55]. The anti-inflammatory action of HM01 has not yet been specifically investigated.

The loss of bone mineral density is a relevant clinical problem in younger cancer patients and associated with increased fracture risk [36]. HM01 improved bone mineral density. Similar to the effect of HM01 on muscle mass, increased GH/IGF-1 signaling might have mediated this treatment effect because IGF-1 induces bone formation [56]. HM01 increases GH and IGF-1 (Pietra C., unpublished data), although we did not re-confirm this in our current study.

We tested a higher dose in order to determine if the strong terminal deterioration of CACS can be counteracted. Because the half-lifetime of HM01 is about 4 h [32], we increased the dosage to 20 mg/kg/day given twice a day, which should result in constantly elevated HM01 blood levels. Despite an apparent increase in food intake compared to the lower dose, HM01 failed to prevent end-stage CACS under our conditions. In this context it needs to be emphasized that it is the major aim of anti-CACS treatment to positively influence the nutritional and general health status during early to mid-stage phases of the disease in order to improve treatment success, quality of life and survival rate. Due to the increasing tumor burden and aggressiveness, virtually all experimental tumor models are characterized by treatment-resistant CACS during the late- and end-stage phase because the tumor itself is usually not treated. Nevertheless, we cannot exclude that the lack of HM01 to prevent end-stage CACS may be linked to ghrelin resistance. Identifying possible neuronal correlates of this treatment resistance, e.g., by terminal c-Fos studies, is difficult because cancer-induced c-Fos expression cannot easily be dissociated from treatment-induced Arc activation. Inflammatory stimuli such as MIC-1 [57], IL-1β [58] and lipopolysaccharide [59] also lead to c-Fos expression in the Arc, in particular in neurons expressing the anorectic neuropeptide α-melanocyte-stimulating hormone (pro-opiomelanocortin (POMC) neurons) [60], while ghrelin stimulates orexigenic neuropeptide Y (NPY) neurons [37]. Further, due to rapid axonal transport, these neuropeptides cannot be detected by standard immunohistochemical studies in the cell bodies. The available transgenic mouse lines that are commonly used to phenotype POMC and NPY neurons (POMC-eGFP and NPY-eGFP mice, eGFP: enhanced green fluorescent protein) [61] cannot be used as tumor recipients for our model because of an incompatible genetic background. Under clinical conditions, treatments of cancer patients last for several months to years allowing much more time for therapeutic interventions against CACS compared to comparatively short treatment periods that are possible in rodent tumor models. Most pre-clinical studies explore anti-CACS therapies in the absence of anti-tumor treatments that would parallel such approaches under clinical conditions. Hence, possible beneficial effects on anti-cancer treatment success are not reflected and possibly underestimated. From this point of view and also taking improvement in quality of life of cancer patients into account, the possible therapeutic benefit cannot only be judged by comparison of end-stage values. In addition to treatment effectiveness, drug safety and tolerability are very important aspects during anti-CACS treatments. Compared to numerous and severe side effects of currently used drugs [62], ghrelin or ghrelin agonists appear to be more suitable. A major concern was a possible enhancement of tumor growth, but in line with other clinical human and rodent studies [23,26,28,29,30,31,63], HM01 did not affect tumor growth in our recent [27] and current studies.

In conclusion, the current study extends our previous demonstration that HM01, as other ghrelin agonists described in literature [28,38] and evaluated in clinical trials [25,64], could be a promising compound for the treatment of CACS. HM01 treatment exerted beneficial effects on energy intake, body weight, fat mass, muscle mass, bone mineral density, and metabolism in a tumor model for inflammatory cancer cachexia. HM01 was effective after oral administration and capable to counteract cachexia without interfering with cytokine or E3 ligase signaling.

## 4. Materials and Methods

### 4.1. Animals and Housing Conditions

Adult male CD2F1 (DBA/1 × balb/c) mice (Charles River, Calco (Lecco), Italy) weighing between 22 and 26 g were used for all behavioral and metabolic experiments involving TB and NTB mice. Animals were kept in a temperature-controlled room (21 ± 1 °C) in 12:12 h light/dark cycling with ad libitum access to tap water and standard chow (890 25 W16, Provimi Kliba, Kaiseraugst, Switzerland). For the testing of the GHS-R agonist HM01 (Helsinn Healthcare SA, Lugano, Switzerland), mice were single housed in plexiglas cages. Mice were handled daily and kept in the cages for at least one week for adaptation. All animal procedures were approved by the Veterinary Office of the Canton of Zurich (license 95/2013, approved 23 May 2014), Switzerland.

### 4.2. Cell Culture and Tumor Model

The C26 tumor model is frequently used for the study of cancer cachexia and has been previously described [35]. Colon-26 tumor cells were kindly provided by Klaske van Norren [47]. Cells were cultured under standard conditions in RPMI 1640 + Glutmax^™^ medium (Gibco^®^—Lifetechnologies^™^, New York, NY, USA) supplemented with 10% fetal bovine serum and 1% penicillin-streptomycin. Confluent C26 Petri dishes (70–80%) were trypsinated and then washed repeatedly with RPMI 1640 to harvest the cells. After confirming viability (≥80%) of the cells with trypan blue, $10^{6}$ cells were inoculated in 250 μL phosphate-buffered saline (PBS) subcutaneously into the right flank under short isoflurane anesthesia.

### 4.3. Effects of HM01 on Food Intake, Body Weight, and Neuronal Activation of the Arc in Healthy, Non Tumor-Bearing Mice

NTB healthy mice were single housed in BIODAQ cages (Research Diets, New Brunswick, NJ, USA) equipped with external food hoppers for the continuous measurement of food intake over 24 h. After 10 days of adaptation to housing conditions, mice were treated daily before dark onset with HM01 (10 mg/kg) diluted in 0.1% methyl-cellulose or methyl-cellulose (controls) via oral gavage for 14 consecutive days. Body weight and food intake were measured daily shortly before treatments. On the final treatment day, animals were anesthetized with pentobarbital (100 mg/kg, intraperitoneal) 2 h after dark onset and perfused with phosphate buffered saline (PBS) for 2 min, followed by 4 min of 4% paraformaldehyde (both kept on ice). Brains were removed, postfixed in 4% paraformaldehyde for 24 h at 4 °C and then cryo-protected in 20% sucrose overnight at 4 °C. Subsequently brains were snap-frozen at −20 °C in hexane before processing for immunohistochemistry (details see section immunohistochemistry). Mice carcasses were kept for the measurement of body composition by computed tomography (CT) scanning.

### 4.4. Effects of C26 Tumor Growth on Food Intake, Body Weight, Body Composition, and Metabolism at Different Time Points after Tumor Induction

In order to identify the effects of C26 tumor growth on food intake, body weight, body composition, metabolism, inflammatory cytokines and muscle proteolytic enzymes at different stages of the cachectic response, we sacrificed C26 TB mice at different time points. The time points were chosen according to pilot experiments. Accordingly, mice were euthanized before the initial loss of body weight (day 9), at the first day when significant body weight loss was reached (day 15) and at the end of experiment, when animals displayed severe body weight loss (day 20).

After 10 days of adaptation to housing conditions, tumor cells were inoculated. Food intake, body weight and tumor size were measured daily shortly before dark onset. Food intake was corrected for spillage and body weight was corrected for tumor weight. For the estimation of tumor weight, tumor length and width were measured daily with a digital caliper. As previously described [65], tumor size was calculated using the equation 0.75 × (0.5 × (length × width${}^{2}$)). A factor of 0.75 was used to correct for overestimation of tumor size when measured through the skin. As confirmed in previous pilot studies tumor volume of 1 cm${}^{3}$ corresponds to a tumor weight of approximately 1 g.

At sacrifice, animals were first placed under short isoflurane anesthesia to collect blood and then injected with pentobarbital before perfusion with PBS for 2 min. Hind limb muscles (gastrocnemius, tibialis anterior, soleus and quadriceps) were dissected, weighed and snap-frozen for the analysis of MuRF-1 and MAFbx gene expression. Total muscle mass was calculated from individual muscle mass.

To investigate the effects of C26 tumor growth on metabolic rate and respiratory gas exchange, we housed the animals in an open-circuit indirect calorimetric system (TSE Phenomaster, TSE Systems GmbH, Bad Homburg, Germany). Respiratory gas exchange (O${}_{2}$ and CO${}_{2}$) was recorded automatically at 17-min intervals throughout the entire experiment. Cages were additionally equipped with internal food hoppers for the manual measurement of food intake. Metabolic parameters were calculated from O${}_{2}$ consumption and CO${}_{2}$ production using the average over 22 h from the 17-min interval recordings. From these averaged values respiratory exchange rate (RER) was calculated as VCO${}_{2}$/VO${}_{2}$. Energy expenditure (EE) calculations were based on the Weir equation: EE (kcal/h) = (3.9 × VO${}_{2}$ + 1.1 × VCO${}_{2}$)/1000. Energy expenditure data in the experiment involving lean mass differences between the two groups were normalized for body weight. Additionally, a telemetric sensor (DSI^™^, New Brighton, MN, USA) was implanted into the intra-peritoneal space to record locomotor activity.

### 4.5. Effects of HM01 on Food Intake, Body Weight, Body Composition, Cytokines, and E3 Ubiquitin Ligases in Tumor-Bearing Mice

Mice were single housed in BIODAQ cages (Research Diets, NJ, USA). Starting from day 9 after tumor induction, mice were treated daily with HM01 (10 mg/kg) via oral gavage until the end of the experiment at day 20. Some animals had to be euthanized before the end of the experiment because they had reached the criteria for the discontinuation of the experiment, based on ethical considerations. In addition, some samples had to be excluded because of insufficient quality (e.g., messenger RNA quality). Body weight and tumor size were measured daily shortly before the treatments. At the end of experiment blood and hind limb muscle were collected as described above.

### 4.6. Effects of HM01 on Metabolic Rate and Respiratory Gas Exchange

Mice were housed in an open-circuit indirect calorimetry system to investigate the effects of HM01 treatment on metabolic rate and respiratory gas exchange as described above. Daily oral treatment was started 10 days after tumor cell inoculation. A higher dose of 2 × 20 mg/kg was used in this experiment. Mice received HM01 or vehicle shortly before dark onset and at the beginning of the light phase. At the same time, food intake, body weight and tumor size were measured.

### 4.7. Body Composition

Total lean mass, fat mass and bone mineral density were measured by quantitative microcomputed tomography (LaTheta LCT-100A scanner, Hitachi-Aloka Medical Ltd., Tokyo, Japan). Sequential 1-mm slice images with a pixel size of 250 × 250 μm were used for calculations using LaTheta software (version 2.10, Hitachi-Aloka Medical Ltd., Tokyo, Japan). Head, lungs, and tail were excluded from the analysis of tissue mass. For the measurement of bone mineral density only the lumbar spine bones were analyzed.

### 4.8. Immunohistochemistry of Arc

Arc sections (20 μm) were cut in a cryomicrotome, thaw-mounted on microscopic glass slides and stored at −20 °C until further processing. Sections were air dried for 1 h, followed by 2 × 5 min rehydration in 0.1% Triton^®^ X-100 in PBS. To block unspecific binding, slides were incubated in blocking solution (PBS containing 0.3% Triton^®^ X-100, 25% Avidin and 1.5% normal goat serum (NGS). The primary antibody (rabbit polyclonal anti-c-Fos (PC38, Merck Millipore, 1:10,000 in PBS) containing 0.3% Triton^®^ X-100 and 25% Biotin solution was applied for 48 h at 4 °C. The secondary antibody (biotinytated goat-anti rabbit, Vector Laboratories, 1:400 in PBS containing 0.3% Triton^®^ X-100 and 1.5% NGS) was applied for 90 min at room temperature. Sections were then incubated in avidin-biotin complex reagent using a kit (ABC Vectastain^®^, Burlingame, CA, USA) for 1 h at room temperature. After 10-min washing in 0.1% Triton^®^ in PBS, followed by a 10-min pretreatment with 0.05 M Tris*HCl, hydrogen-peroxide-enhanced diaminobenzadine (DAB, Pierce, Rockford, IL, USA) was added for 4–8 min to visualize the immunoreactivity. Sections were then washed 3 × 5 min in 0.05 M Tris*HCl, 0.1% Triton^®^ in PBS, and PBS only. Before cover slipping, slides were dehydrated by immersion in different concentrations of ethanol (50%, 75%, 95%, 100% for 5 min each) followed by 5 min in xylol. c-Fos positive cell nuclei were quantified in a blinded manner from three slices of the Arc between Bregma −1.90 mm and Bregma −2.50 mm under a light microscope (Zeiss Imager Z2 microscope, Carl Zeiss AG, Oberkochen, Germany). Cell counts from these three slices were averaged per animal and used for the calculation of group means. Photomicrographs were taken at 20× magnification, using a digital camera system (Zeiss Axiocam, Carl Zeiss AG, Oberkochen, Germany).

### 4.9. Muscle PCR Analysis

For the gene expression analysis of MuRF-1 (Trim63) and MAFbx (Fbxo32), the samples were snap-frozen in liquid nitrogen immediately after dissection and stored at −80 °C. RNA was extracted from 10 mg muscle tissue using ReliaPrep^®^ Tissue Miniprep System (Promega, Madison, WI, USA). A total of 0.75 ng complementary DNA (cDNA) (High capacity RNA to cDNA Kit, Applied Biosystems) was used for PCR reaction including 10 μL of ddPCR Supermix for Probes (BioRad) and 1 μL of each 20× TaqMan gene expression assay (Applied Biosystems, Carlsbad, CA, USA). The following Taqman assays were used: Mm00499523_m1 for MAFbx and Mm01185221_m1 for MuRF-1. Quantitative polymerase chain reaction assays were performed using duplexed FAM and VIC TaqMan assays in a droplet digital PCR system (QX100; Bio-Rad Laboratories, Inc., Hercules, CA, USA) as previously described [66].

### 4.10. Plasma Cytokines

Blood collected in EDTA tubes (Sarstedt, Nümbrecht, Germany) was immediately centrifuged at 2000× *g* (4 °C, 20 min). The plasma was then aliquoted and stored at −80 °C until further processing. MIC-1 plasma levels were analyzed using Mouse/Rat GDF-15 Quantikine ELISA Kit (R&D Systems, USA) according to the manufacturers’ instructions. IL-6 plasma values were analyzed using the electrochemiluminescent V-PLEX Proinflammatory Panel 1 (mouse) Kit (MSD, Gaithersburg, MD, USA).

### 4.11. Data Analysis and Statistics

All data are expressed as mean ± standard error of the mean. Unpaired Student’s *t*-test (two-sided) was used for comparison between two groups. Statistical comparisons between multiple groups were performed using one-way ANOVA followed by Tukey’s post-hoc test. Data were tested for normality using Kolmogorov–Smirnov test. For all statistical tests, a *p*-value lower than 0.05 was considered significant. Data were analysed using Prism GraphPad 7.0.

## Abbreviations

CACS	Cancer anorexia-cachexia syndrome
C26	Colon-26 tumor model
IL-6	Interleukin-6
TNF-α	Tumor necrosis factor-α
IGF-1	Insulin-like growth factor-1
GH	Growth hormone
GHS-R	Growth hormone secreatgogue receptor
GHS-R1a	Growth hormone secreatgogue receptor 1a
MURF-1	Muscle RING-finger protein-1
MAFbx	Muscle atrophy F-box / atrogin-1
MIC-1	Macrophage-inhibitory cytokine-1
TB	Tumor-bearing
NTB	Non tumor-bearing
PBS	Phosphate-buffered saline
NGS	Normal goat serum
POMC	Pro-opiomelanocortin
NPY	Neuropeptide Y
eGFP	Enhanced green fluorescent protein
cDNA	Complementary DNA
